# Supplementary material for: The role of Rnf in ion gradient formation in Desulfovibrio alaskensis
Source: PeerJ. 2016 Apr 14;4:e1919. doi: 10.7717/peerj.1919 (PMC4841214; doi:10.7717/peerj.1919)
Supplement: Supplemental Information 1 — This is a complete file with all of the supplementary figures. [file peerj-04-1919-s001.docx]

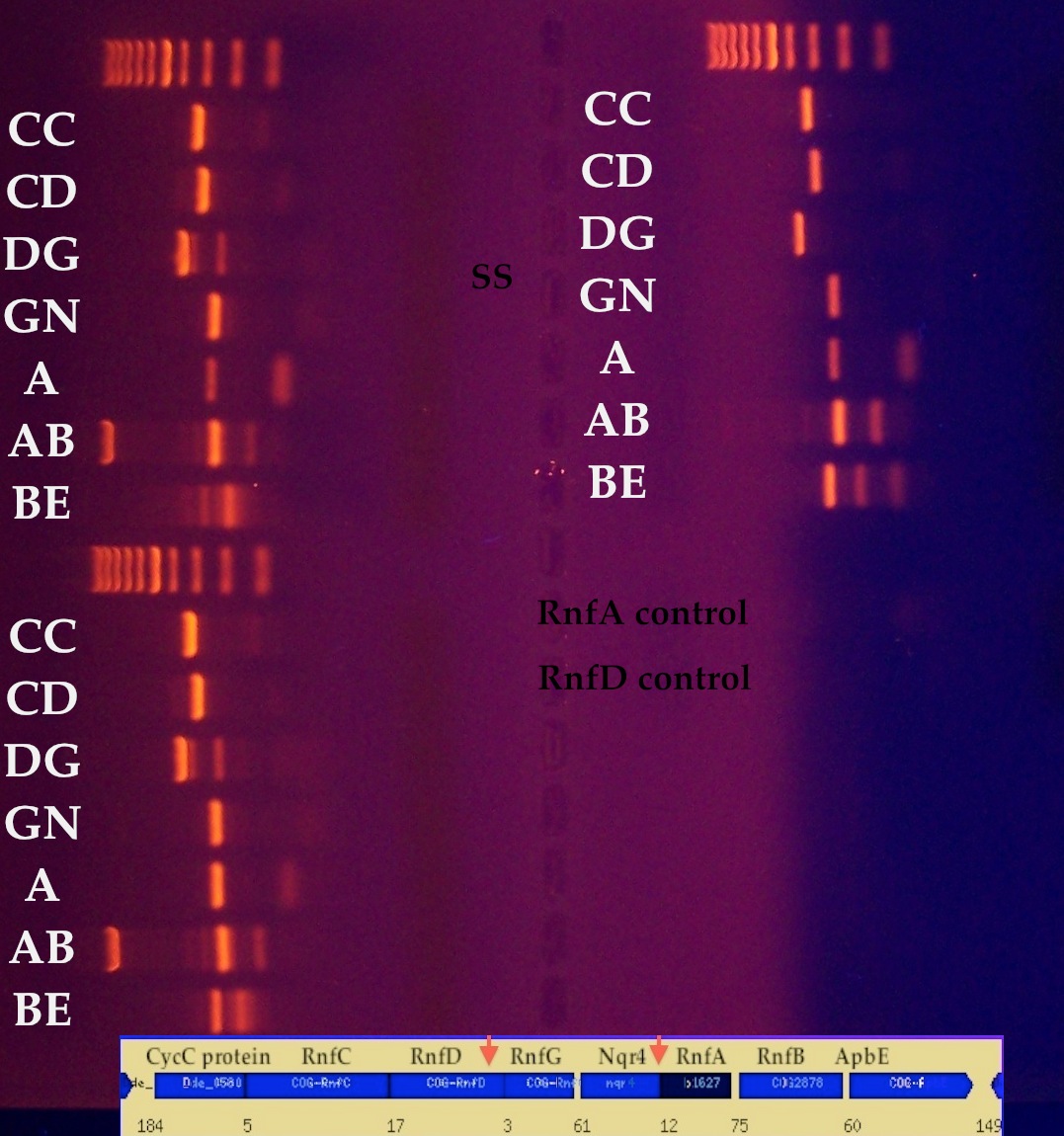


Figure S1. Results from PCR analysis showing the amplification of gene intervening regions on the transcripts. On the left of the gel, results from the rnfA mutant on on top and the rnfD mutant are underneath. The results from the parent strain are on the right side of the gel. GN corresponds to the rnfG-rnfE gap and A corresponds to the rnfE-rnfA gap represented by primers in Table 1.


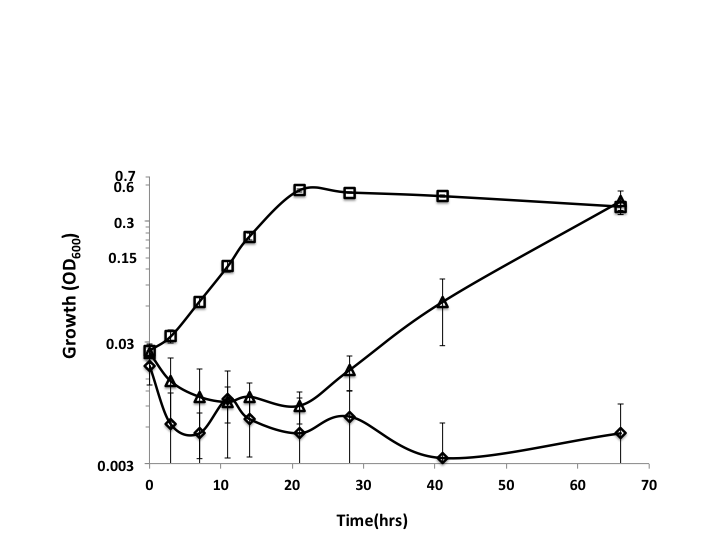


Figure. S2. Growth curve on H_2_/SO_4_^2-^ for *D. alaskensis* G20 parent strain (□), the *rnfA* (◇) and the *rnfD* (△) mutant. Standard deviation is shown.


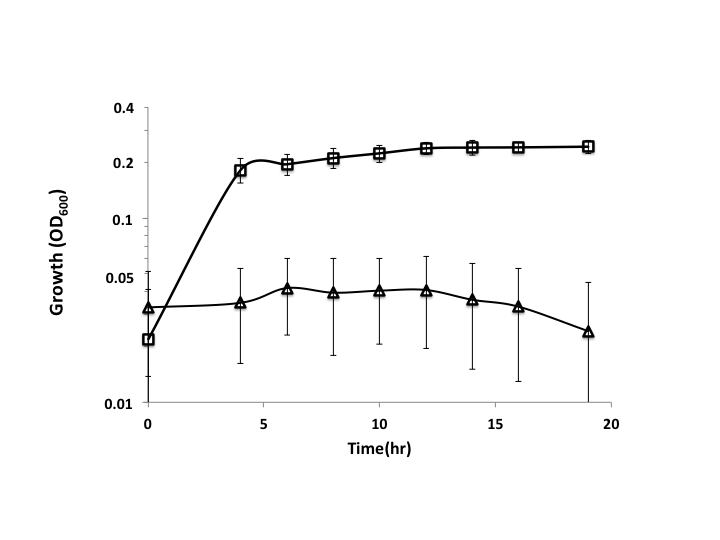


Figure. S3. Growth curve on Ethanol (25mM)/SO_4_^2-^ (10 mM) for *D. alaskensis* G20 parent strain (□) and the *rnfA* (△) mutant. Standard deviation is shown.


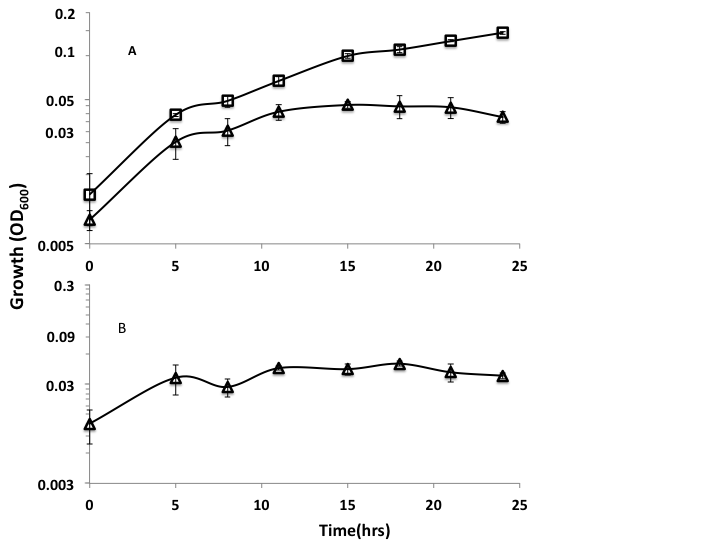


Figure S4. First day of growth curves on H_2_/SO_4_^2-^ for *D. alaskensis* G20 parent strain (□) and *rnfD* mutant (△). (A) One set of cultures was incubated in the basal media described in methods (B) and the other set had 0.05% casamino acids added. Standard deviation is shown.


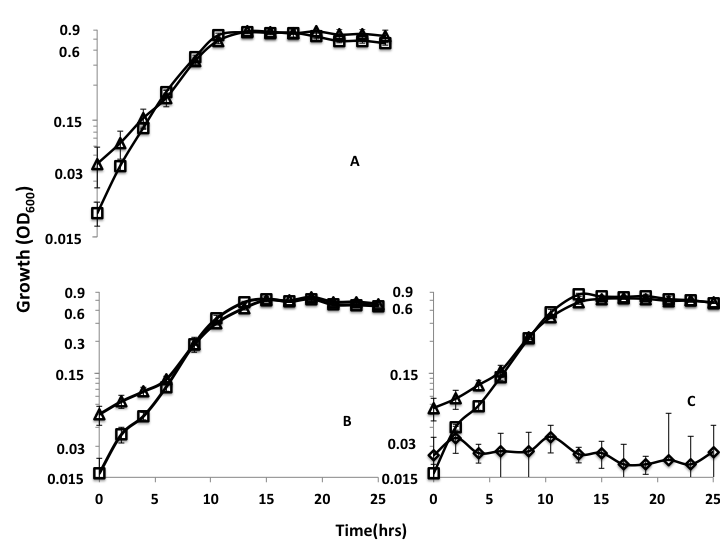


Figure S5. Growth curves on Lactate/SO_4_^2-^ for *D. alaskensis* G20 parent strain (A), the *rnfA* (B) and the *rnfD* (C) mutant. One set of cultures was incubated with ETH 2120 (20 µM) (△) and the other without (□) . In plot C, the lack of growth of the *rnfD* mutant with 5uM TCS is shown (◇). Standard deviation is shown.


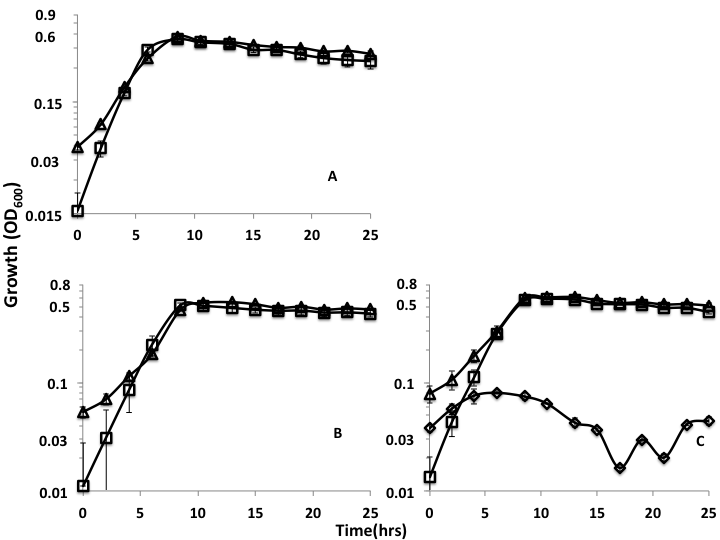


Figure S6. Growth curves on Lactate/SO_3_^2-^ for *D. alaskensis* G20 parent strain (A), the *rnfA* (B) and the *rnfD* (C) mutant. One set of cultures was incubated with ETH 2120 (20 µM) (△) and the other without (□). In plot C, the lack of growth of the *rnfD* mutant with 5uM TCS is shown (◇). Standard deviation is shown.


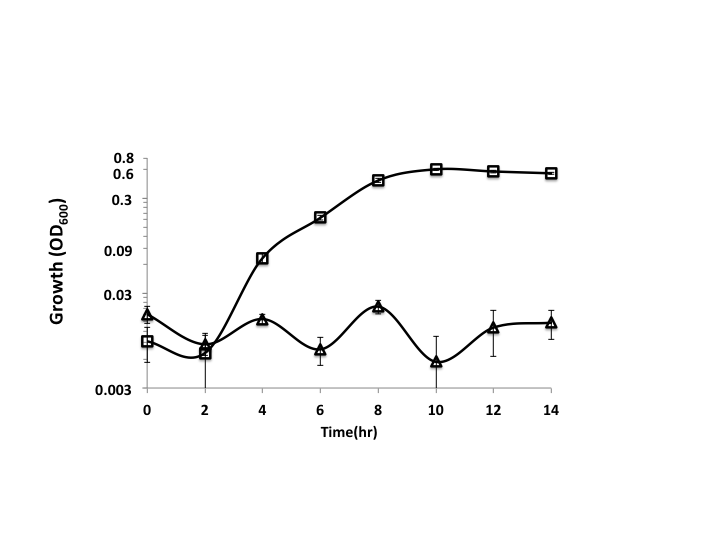


Figure S7. Growth curves on Lactate/SO_3_^2-^ for *D. alaskensis* G20 *rnfA* mutant. One set of cultures was incubated with TCS (20 µM) (△) and the other without (□). Standard deviation is shown.
