# Supplementary figures and images for: The role of Rnf in ion gradient formation in Desulfovibrio alaskensis
Source: PeerJ. 2016 Apr 14;4:e1919. doi: 10.7717/peerj.1919 (PMC4841214; doi:10.7717/peerj.1919)

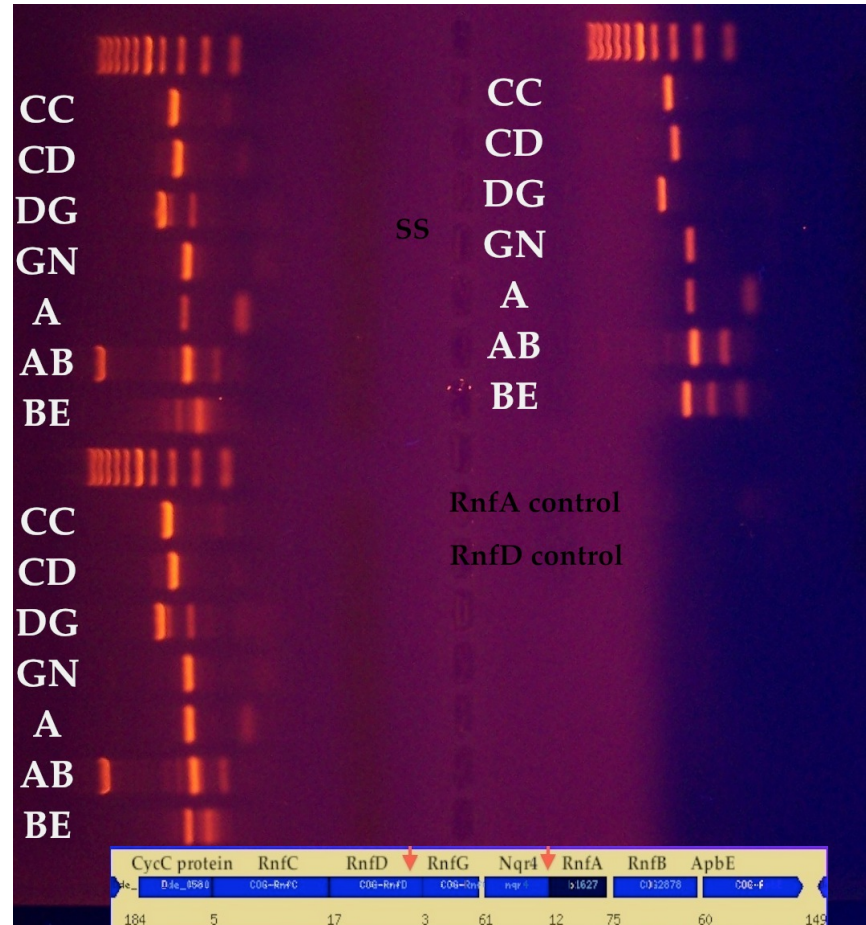

Supplement: Supplemental Information 2 — Results from PCR analysis showing the amplification of gene intervening regions on the transcripts. On the left of the gel, results from the rnfA mutant on top and the rnfD mutant are underneath. The results from the parent strain are on the right side of the gel. GN corresponds to the rnfG-rnfE gap and A corresponds to the rnfE-rnfA gap represented by primers in Table 1. [file peerj-04-1919-s002.pdf]

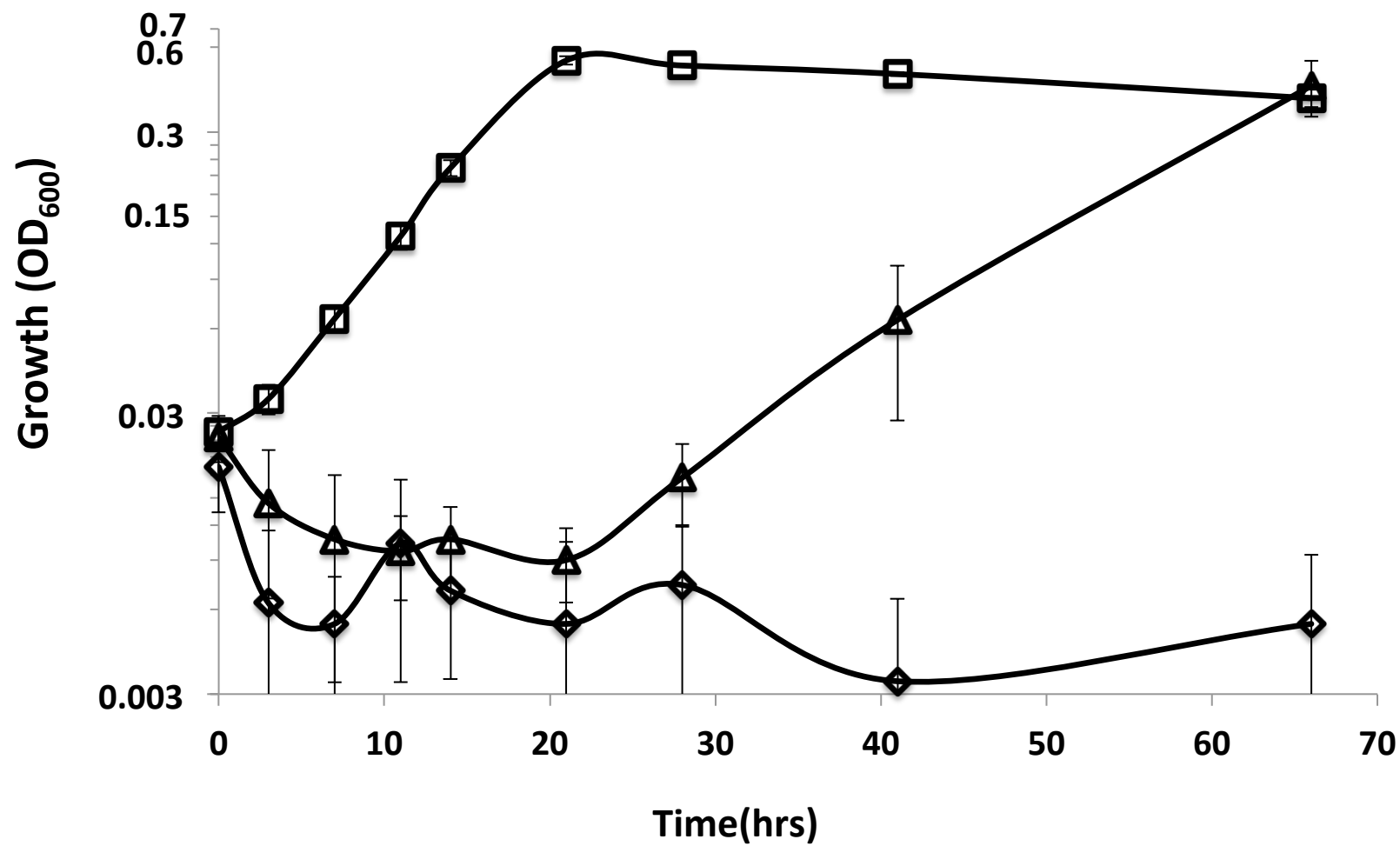

Supplement: Supplemental Information 3 — Growth curve on ${\mathrm{H}}_{2}/{\mathrm{SO}}_{4}^{2-}$H2∕SO42− for D. alaskensis G20 parent strain (squares), the rnfA (diamonds) and the rnfD (traingles) mutant. Standard deviation is shown. [file peerj-04-1919-s003.pdf]

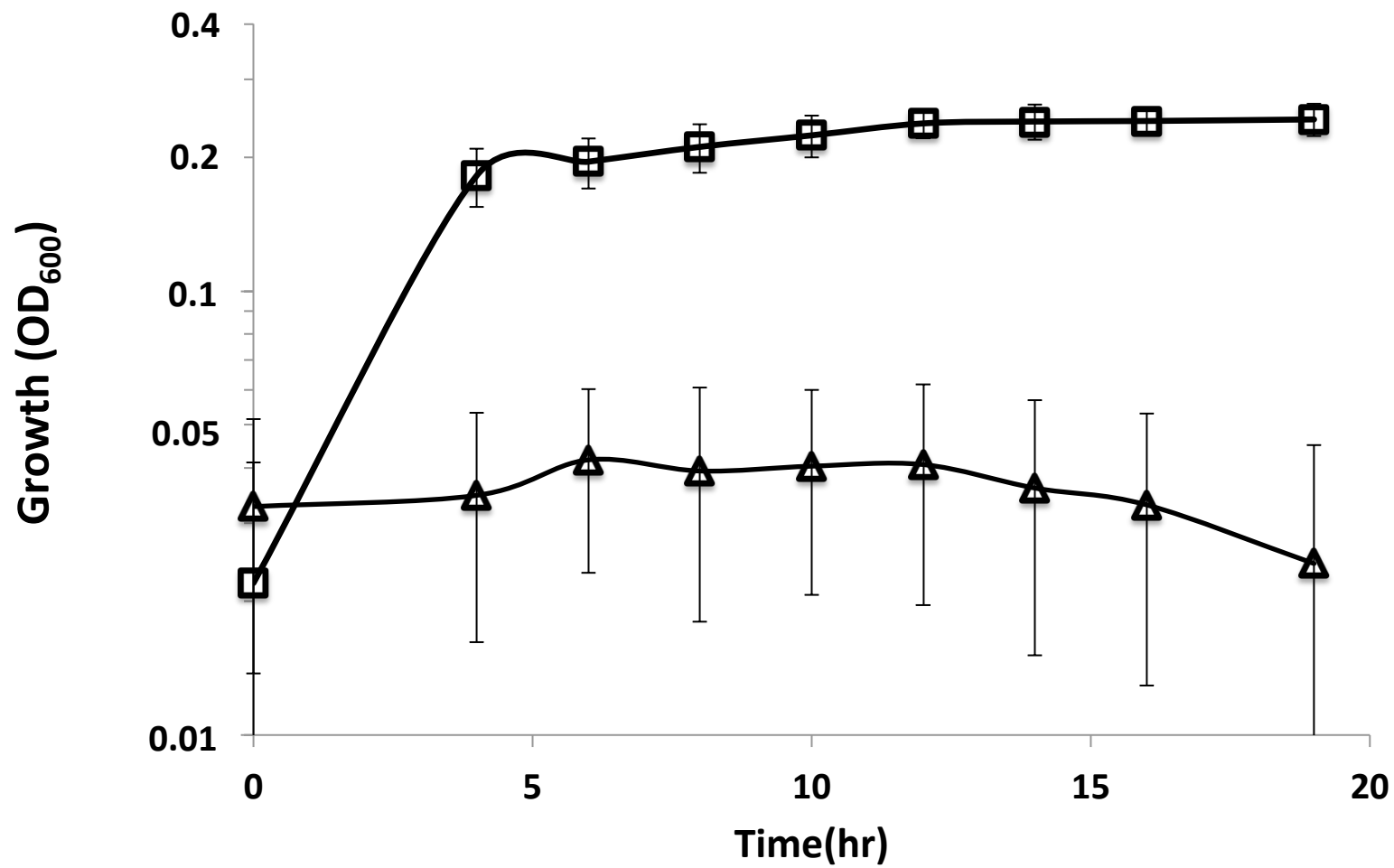

Supplement: Supplemental Information 4 — Growth curve on Ethanol $(25\hspace*{1em}\mathrm{mM})/{\mathrm{SO}}_{4}^{2-}(10\hspace*{1em}\mathrm{mM})$25mM∕SO42−10mM for D. alaskensis G20 parent strain (squares) and the rnfA (traingles) mutant. Standard deviation is shown. [file peerj-04-1919-s004.pdf]

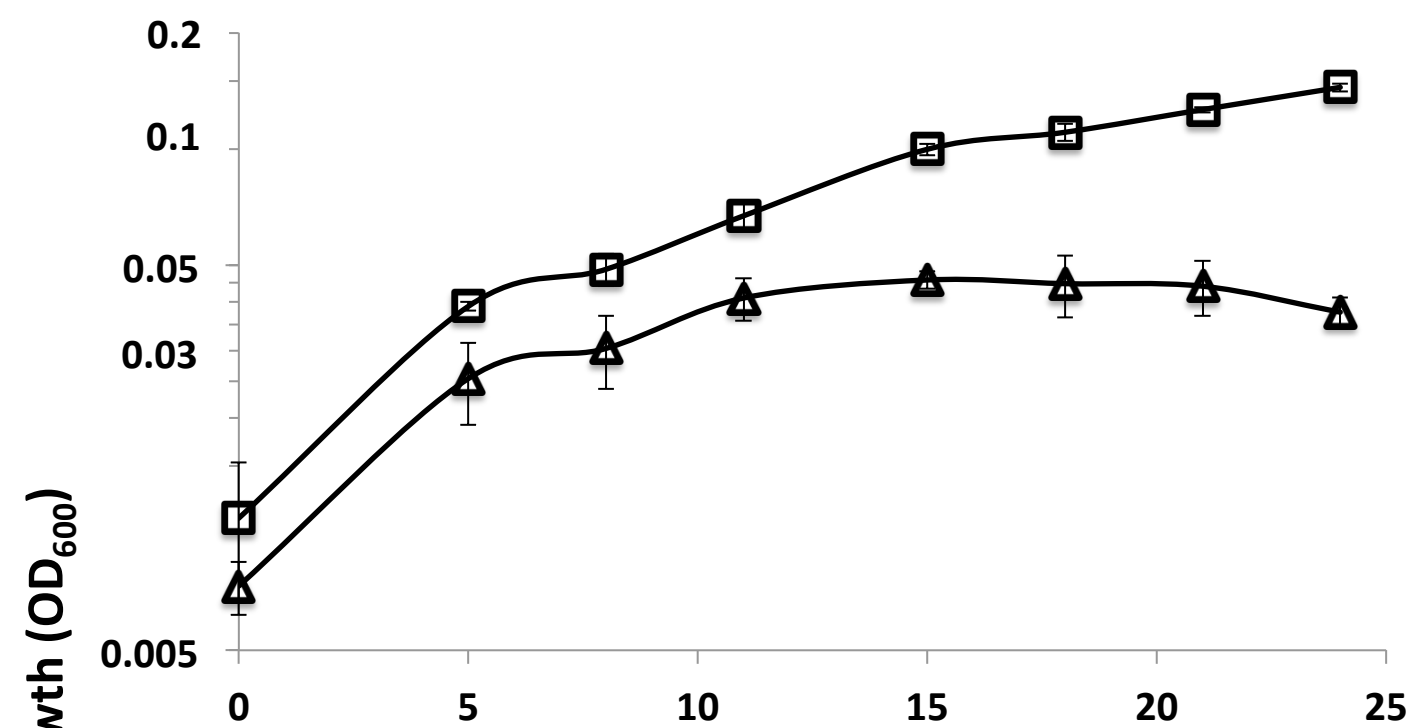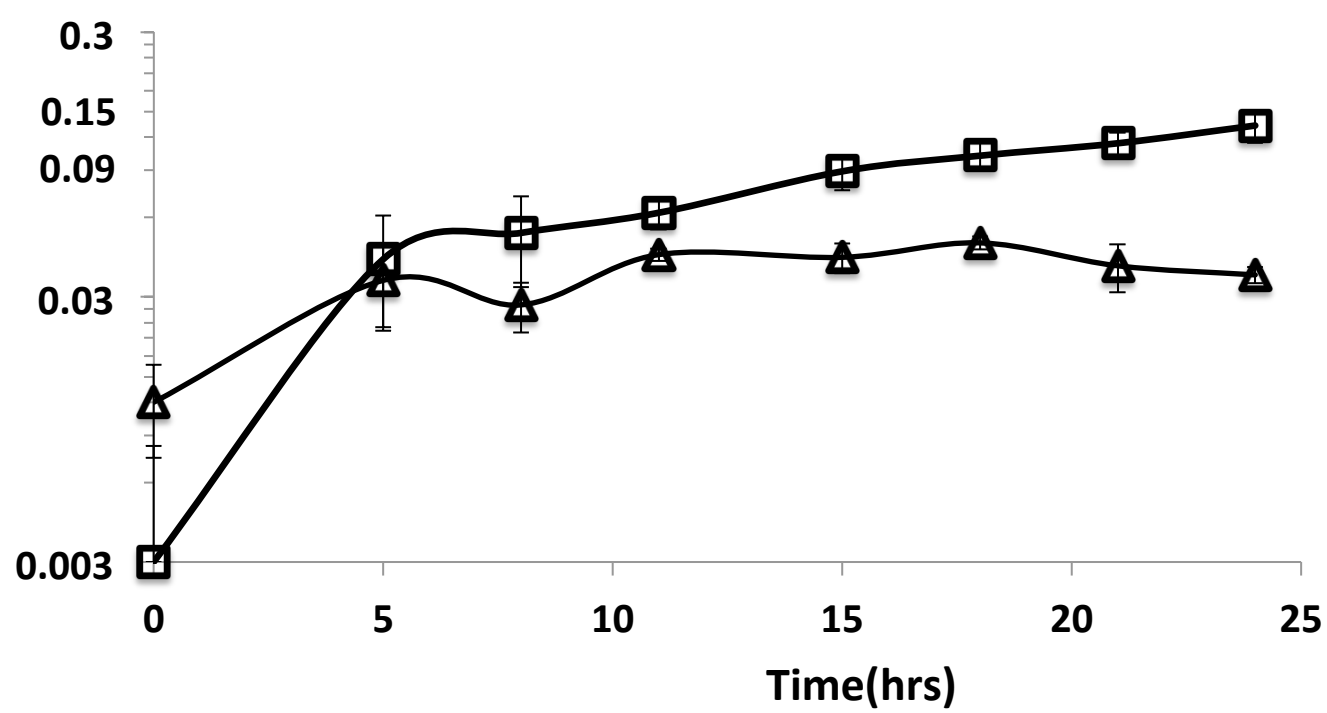

Supplement: Supplemental Information 5 — First day of growth curves on ${\mathrm{H}}_{2}/{\mathrm{SO}}_{4}^{2-}$H2∕SO42− for D. alaskensis G20 parent strain (□) and rnfD mutant (△). (A) One set of cultures was incubated in the basal media described in methods (B) and the other set had 0.05% casamino acids added. Standard deviation is shown. [file peerj-04-1919-s005.pdf]

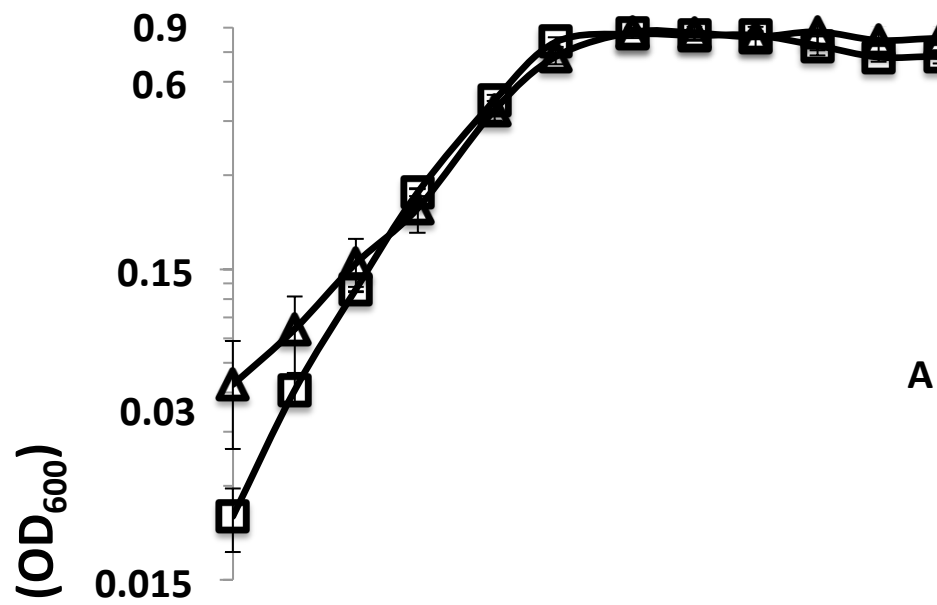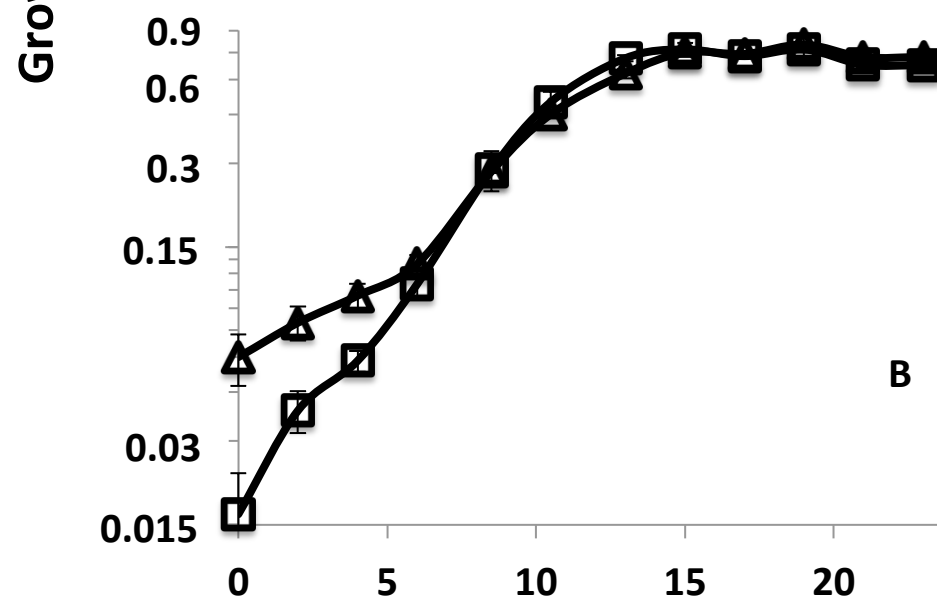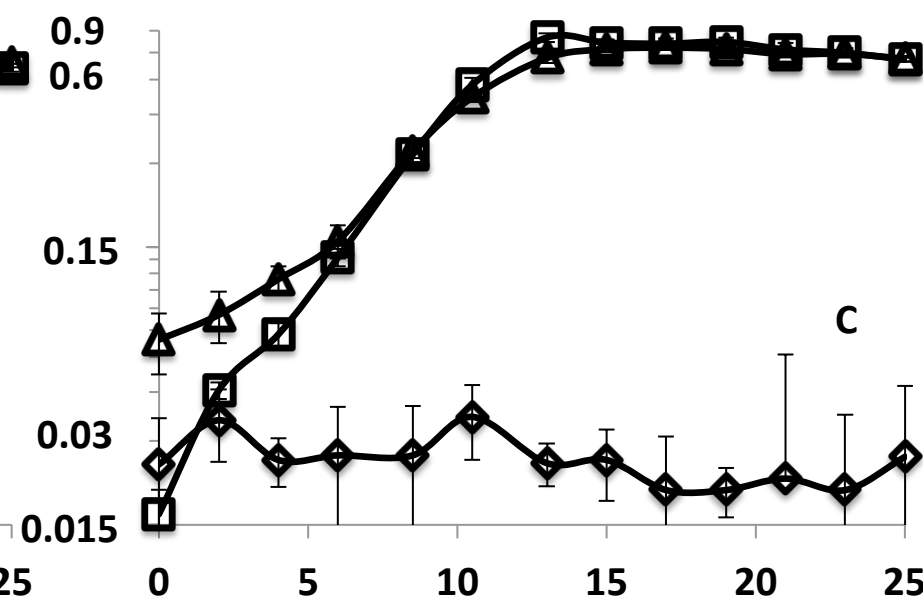

Supplement: Supplemental Information 6 — Growth curves on $\text{Lactate}/{\mathrm{SO}}_{4}^{2-}$Lactate∕SO42− for D. alaskensis G20 parent strain (A), the rnfA (B) and the rnfD (C) mutant. One set of cultures was incubated with ETH 2120 (20 µM) (triangles) and the other without (squares). In plot C, the lack of growth of the rnfD mutant with 5uM TCS is shown (diamonds). Standard deviation is shown. [file peerj-04-1919-s006.pdf]

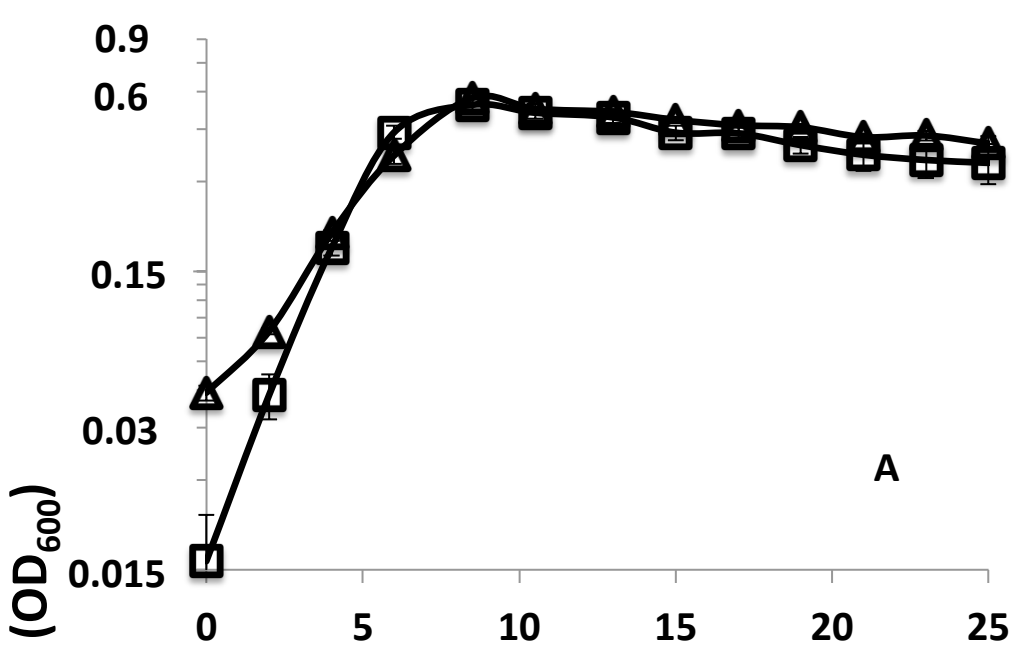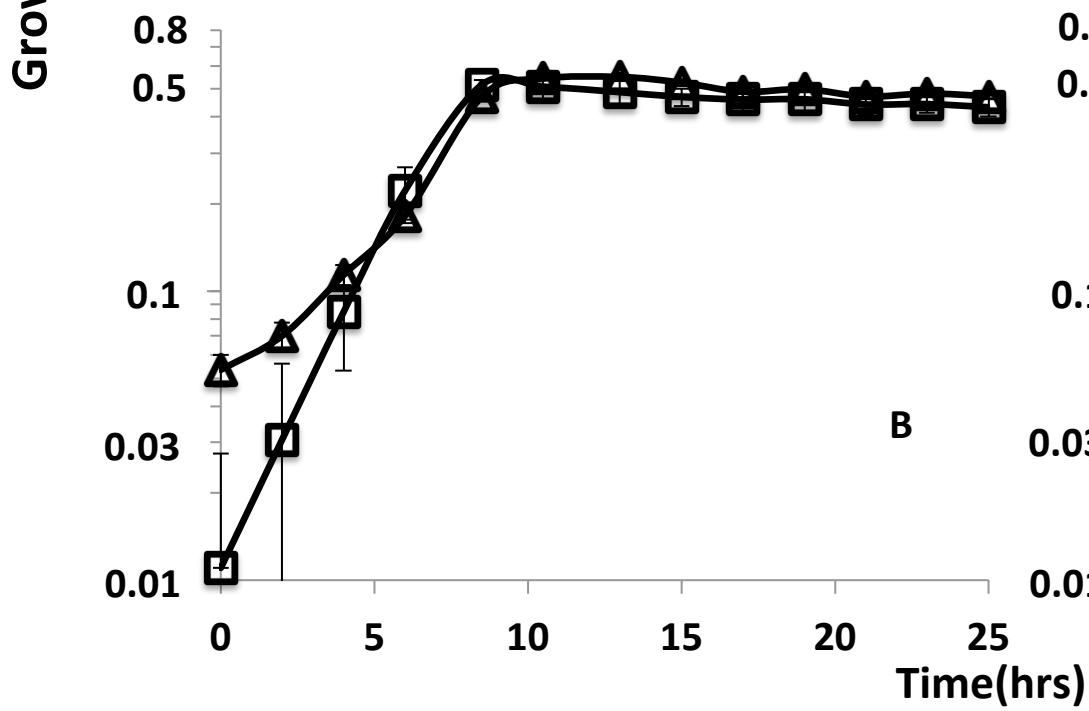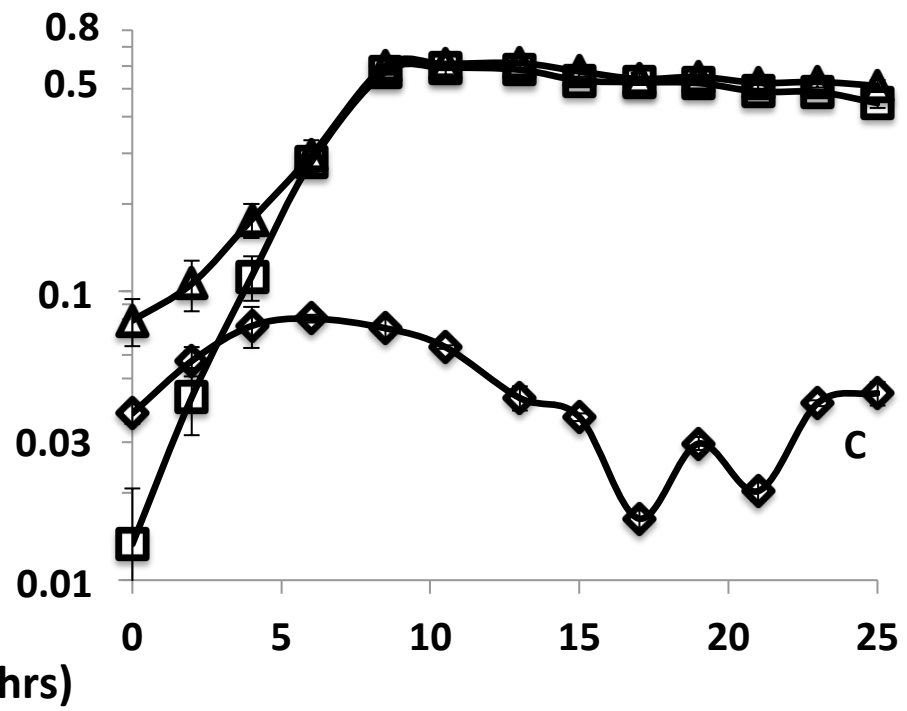

Supplement: Supplemental Information 7 — Growth curves on $\text{Lactate}/{\mathrm{SO}}_{3}^{2-}$Lactate∕SO32− for D. alaskensis G20 parent strain (A), the rnfA (B) and the rnfD (C) mutant. One set of cultures was incubated with ETH 2120 (20 µM) (triangles) and the other without (squares). In plot C, the lack of growth of the rnfD mutant with 5uM TCS is shown (diamonds). Standard deviation is shown. [file peerj-04-1919-s007.pdf]

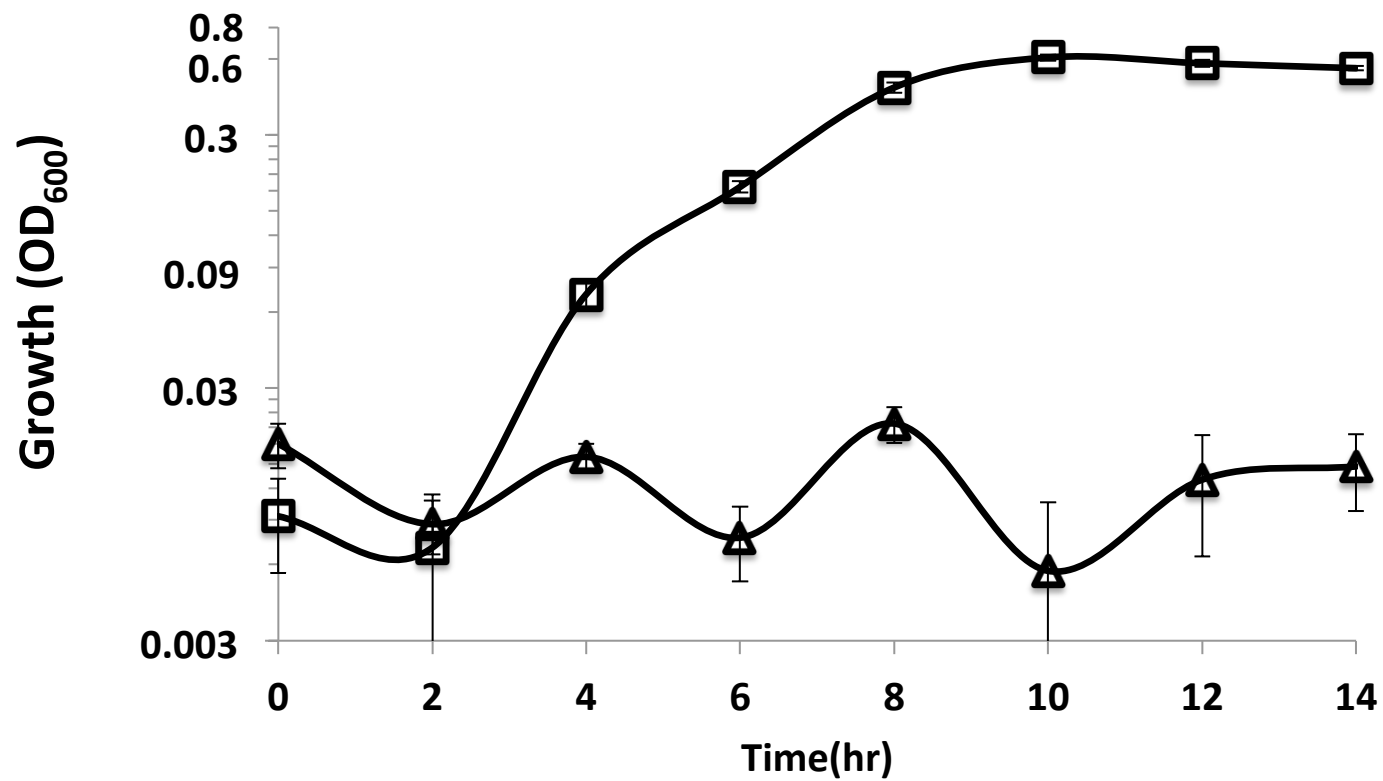

Supplement: Supplemental Information 8 — Growth curves on Lactate/SO32- for D. alaskensis G20 rnfA mutant. One set of cultures was incubated with TCS (20 µM) (triangles) and the other without (squares). Standard deviation is shown. [file peerj-04-1919-s008.pdf]
